# Intestinal SURF4 in dyslipidaemia and female-specific metabolic disorders: insights from rats with polycystic ovary syndrome

**DOI:** 10.3389/fnut.2025.1644496

**Published:** 2025-09-17

**Authors:** Yating Xu, Yu Si, Li Ning, Ruyue Wang, Hua Ma, Xiu Li, Hongting Zhao, Qingling Ren

**Affiliations:** ^1^Nanjing University of Chinese Medicine, Nanjing, China; ^2^Jiangsu Provincial Traditional Chinese Medicine Obstetrics and Gynecology Reproductive Clinical Medical Innovation Center, Nanjing, China

**Keywords:** circulating lipids, polycystic ovarian syndrome, SURF4, lipid metabolism, LDL, HDL, TG

## Abstract

**Background:**

Polycystic ovary syndrome (PCOS) is a complex endocrine disorder characterized by hyperandrogenism, ovulatory dysfunction, and metabolic disturbances, including dyslipidaemia. Recent studies have suggested that intestinal surfeit locus protein 4 (SURF4) contributes to elevated serum PCSK9 levels and subsequent lipid accumulation, with evidence of sex-specific differences in its expression and regulatory effects. This study aimed to investigate the involvement of intestinal SURF4 in the pathogenesis of PCOS and its potential contribution to metabolic lipid disturbances, as well as to explore lipid-PCOS associations through Mendelian randomization (MR) analysis.

**Methods:**

This research established two rat models of PCOS: one by administering letrozole in combination with a high-fat diet (Model, *n* = 5) and another by subcutaneous injection of DHEA (DHEA, *n* = 5). Intestinal SURF4 expression levels were assessed using immunohistochemistry and quantitative polymerase chain reaction. Quantitative serum lipid profiles and androgen levels evaluated the metabolic and hormonal alterations associated with PCOS. Group differences were assessed using ANOVA with post-hoc tests. Mendelian randomization (MR) analyses were conducted to assess the causal relationship between genetic lipid traits and PCOS risk, using data from 10,074 PCOS cases and 103,164 controls.

**Results:**

The PCOS rat model exhibited significant upregulation of intestinal SURF4 accompanied by dyslipidaemia and elevated androgen levels. Elevated androgen levels may regulate intestinal SURF4 expression, contributing to disturbances in lipid metabolism. MR analyses indicated that PCOS leads to serum lipid abnormalities rather than vice versa.

**Conclusion:**

These findings suggest that intestinal SURF4 may serve as a potential intervention target for improving lipid metabolic disorders associated with PCOS.

## 1 Introduction

Polycystic ovary syndrome (PCOS) is a multifaceted metabolic disorder affecting women from puberty to reproductive age, diagnosed through NIH criteria (requiring hyperandrogenemia and ovarian dysfunction) or Rotterdam criteria (requiring at least two of three traits: hyperandrogenemia, ovarian dysfunction, and polycystic ovarian morphology), and affects approximately 6–10% of women of reproductive age (1). PCOS is clinically heterogeneous across populations and is associated with a wide range of comorbidities, including hirsutism, acne, irregular menstruation, infertility, and metabolic disorders. Notably, PCOS is commonly associated with hyperlipidaemia, obesity, and impaired glucose tolerance (2), predisposing individuals to complications, such as type 2 diabetes (3, 4) and hypertension (5), thereby posing a substantial threat to women’s health (6). Particularly in adolescents, the prevalence of lipid abnormalities is higher among patients with PCOS compared to those without the condition (7).

Epidemiological investigations have revealed a complex relationship between PCOS and hyperlipidaemia (8). Initial cross-sectional studies showed that women with PCOS had a higher prevalence of metabolic disorders, including significantly increased waist-to-hip ratios, triglycerides (TG), and low-density lipoprotein (LDL) levels, compared to healthy controls (9). Subsequent clinical studies have shown that increased LDL levels constitute the primary lipid abnormality in women with PCOS and are not directly associated with obesity (9). Although a 2013 study suggested no statistically significant difference in the prevalence of hyperlipidaemia between patients with PCOS and individuals without (10), a subsequent follow-up study spanning over 10 years revealed an increase in prevalence from 52.3 to 64.3% (11).

Dyslipidaemia influences the pathological progression of PCOS. In clinical practice, monitoring the body mass index (BMI) and lipid profile has become the standard method for assessment (1). Effective lipid management is essential for promoting preconception health in patients with PCOS. In reproductive medicine, lipid metabolic markers such as TG, high-density lipoprotein (HDL), and LDL have emerged as important determinants of early outcomes of *in vitro* fertilization/intracytoplasmic sperm injection (12). Hyperlipidaemia negatively affects embryo quality, endometrial tolerance, and in vitro fertilization outcomes, particularly in normal-weight patients with PCOS (13). Medications, such as omega-3, roflumilast, and metformin, exhibit efficacy in alleviating PCOS symptoms and in considerably reducing circulating lipid levels (14–17). A randomized, double-blind, placebo-controlled study revealed substantial improvements in clinical and biochemical abnormalities, as well as ovarian function, in patients with PCOS with high LDL levels following statin treatment (18).

Metabolic syndromes, such as obesity, hyperandrogenaemia, and insulin resistance (IR) are associated with PCOS and significantly influence dyslipidaemia (19). Predicting the risk of developing metabolic syndrome in PCOS often involves the assessment of lipid parameters, such as HDL and TG levels (20). Numerous studies have noted distinct dyslipidaemia patterns among patients with PCOS, with higher levels of LDL in those with IR and higher levels of TG in those with glucose abnormalities (21). Other studies have identified that varying lipid profiles are associated with different androgen levels, underscoring the important role of androgens in hyperlipidaemia (22). However, mild hypercholesterolemia does not directly affect hormone levels in PCOS; instead, it is associated with IR (23).

Although observational studies have revealed relationships between PCOS and lipids (24, 25), the extent to which these correlations are influenced by potential biases and confounders remains unclear. Therefore, exploring the multifaceted relationships between PCOS and dyslipidaemia is critical for clinical management and future studies (26).

Surfeit locus protein 4 (SURF4) encodes a conserved integral membrane protein. SURF4 is a transporter receptor in the ER membrane that promotes secretion of celiac and VLDL from intestinal epithelial cells and hepatocytes. Genome-wide SURF4 knockout mice developed reproductive toxicity and survived for no more than 2 days (27). Previous studies have focused on the role of hepatic SURF4 in the regulation of circulating lipids. SURF4 is an efficient COPII cargo receptor that partners with SAR1B and selectively interacts with APOB and APOA1 containing lipoproteins, plays a key role in lipoprotein transport. Acute inactivation of hepatic SURF4 leads to an almost complete depletion of plasma lipids and lipoproteins in mice, thereby protecting them from PCSK9 plus diet-induced atherosclerosis (28). Hepatic SURF4 knockout mice showed downregulation of lipids without substantial sex differences, which may exert pro-LDL degradation by promoting proprotein convertase subtilisin/kexin type 9 (PCSK9) secretion (29). The small intestine plays a pivotal role in dietary lipid absorption by mediating the uptake of TGs, total cholesterol (TC), and phospholipids (PLs). These lipids are subsequently assembled into chylomicrons or HDL and secreted into the bloodstream via the mesenteric lymphatic vessels or portal veins. This process supports the systemic lipid requirements and maintains metabolic homeostasis (30). In contrast to previous SURF4 studies in the liver, the absence of intestinal SURF4 led to the accumulation of ingested lipids in the intestine, which in turn triggered a decrease in circulating lipid levels (31). Functional changes in intestinal SURF4 knockdown were considerably different between female and male mice (32).

In the present study, we first measured intestinal SURF4 levels in hyperlipidemic PCOS rats and assessed their correlation with serum levels of PCSK9, LDL, HDL, TG, and TC. The potential causal relationship between circulating lipid levels and PCOS was further investigated using a comprehensive two-sample Mendelian randomization (MR) analysis. In this study, we explored intestinal SURF4 changes and their possible correlation with circulating lipid changes in rats with PCOS using a female rats model that developed elevated androgens. We employed two models: the letrozole-induced high-fat diet PCOS rat model, which mimics disease-related hyperandrogenemia, and the dehydroepiandrosterone (DHEA) treated PCOS rat model, which induces exogenous hyperandrogenemia. These models were used to analyze the role of SURF4 in hormone and high-fat interventions.

## 2 Materials and methods

### 2.1 Experimental animals

Female Sprague–Dawley 3-week-old rats (license No. scxk (Jing) 2021-0011) were purchased from Charles River Laboratories. Rats were reared in the SPF barrier system. The facility operated with restricted access and standard gowning procedures. Animal rooms were supplied with HEPA-filtered air (≥99.97% efficiency for 0.3 μm particles) under positive-pressure ventilation, providing 10–15 air changes per hour. The temperature was controlled at 22 ± 2 °C with relative humidity maintained at 50–60%. Animals were housed in individually ventilated cages (IVCs), each supplied with HEPA-filtered air. Bedding, feed, and drinking water were sterilized prior to use. The health status of the colony was routinely monitored to ensure SPF conditions. The protocol was evaluated and received approval from the animal ethics committee at Nanjing University of Traditional Chinese Medicine, under the approval code 202204A054 and 2024DW-095-01.

After a 3-day adaptation period during which the animals were acclimatized to their surroundings, the rats were randomly divided into Control and Model groups (*n* = 5 per group). Animal sample sizes (*n* = 5 per group) were determined based on previous studies and pilot experiments, aiming for 80% power to detect expected differences in serum androgen and lipid levels at *α* = 0.05. Throughout the 4-week experimental period, the Control group rats were provided with standard chow and received daily oral administration of 1% sodium carboxymethyl cellulose. Rats were fed a standard rodent chow diet (Jiangsu Xietong Pharmaceutical Bio-engineering Co., Ltd., Product No. 1010086). The diet was formulated from chicken meal, fish meal, soybean meal, corn, wheat flour, bran, vegetable oil, and vitamin/mineral premix. Its proximate composition included crude protein ≥18%, crude fat ≥4%, crude fiber ≤5%, ash ≤8%, calcium 1.0–1.8%, and total phosphorus 0.6–1.2%, with adequate essential amino acids and vitamins (e.g., vitamin A ≥ 7,000 IU/kg, vitamin E ≥ 60 IU/kg). Conversely, the rats in the Model groups were supplied with a high-fat diet (sourced from Research Diets, Inc., New Brunswick, NJ, USA) and were orally administered letrozole (sourced from Aladdin, L129473) continuously over a 30-days period. Letrozole (1 mg/kg/day) was administered orally, suspended in 1% sodium carboxymethyl cellulose (CMC-Na) due to its poor water solubility. The rats in the DHEA group were administered subcutaneous injections of DHEA (sourced from Aladdin, D106380) at a dose of 60 mg/kg/day for 21 consecutive days.

Each rat weight was recorded every day. The estrous cycle of female rats was monitored daily for 14 consecutive days following the experimental period (*n* = 5 per group). Vaginal smears were collected each morning using a sterile saline lavage (100 μL), aspirated back into a pipette, and transferred onto clean glass slides. Smears were air-dried and stained with 0.1% toluidine blue for 1–2 min, rinsed with distilled water, and air-dried again. Slides were examined under a light microscope at 20 × magnification. Cell types were classified based on standard cytological criteria: Proestrus (P): Predominantly nucleated epithelial cells. Estrus (E): Predominantly cornified epithelial cells. Metestrus (M): A mixture of leukocytes, cornified, and nucleated epithelial cells. Diestrus (D): Predominantly leukocytes. The stage of the estrous cycle was recorded daily to assess cycle regularity.

Finally, all rats were euthanized after fasting for 12 h and anesthetized with 2% sodium pentobarbital (40 mg/kg). Intestinal tissues were collected, and a portion was fixed in commercial 4% paraformaldehyde (PFA; Biosharp, Cat. No. BL539A) at room temperature for 24 h, followed by routine processing for histological analysis. The remaining fresh tissues were treated with liquid nitrogen and stored in a – 80 °C refrigerator for further experiments. Blood was collected from the abdominal aorta, and serum was centrifuged at 3000 rpm and 4 °C for 15 min. Data are presented as mean ± standard error of the mean (SEM). *p < 0.05, **p < 0.01, ****p < 0.0001.

### 2.2 ELISA assay

To quantify rat serum hormones (LH: H206-1-2, FSH: H101-1-2, Testo: H090-1-2), PCSK9 (H430-1), TG (A110-1-1), TC (A111-1-1), HDL (A112-1-1) and LDL (A113-1-1), we utilized commercially available assay kits (Nanjing Jiancheng Bioengineering Institute) along with a biochemical analyzer, intra-assay CV < 10% and inter-assay CV < 12%. All measurements were conducted in accordance with the manufacturer’s instructions.

### 2.3 Immunohistochemistry

Immunohistochemistry was performed to detect the expression of SURF4 in tissue samples. Tissue sections were deparaffinized in xylene and rehydrated through graded ethanol solutions. Antigen retrieval was conducted by heating the sections in sodium citrate buffer (pH 6.0) at 95 °C for 20 min. Endogenous peroxidase activity was quenched by incubating the sections with 3% hydrogen peroxide for 10 min at room temperature.

After blocking with 5% normal goat serum for 1 h, the sections were incubated overnight at 4 °C with an anti-SURF4 primary antibody (1:300, Abclonal A16744). The next day, sections were washed with PBS and incubated with a biotinylated secondary antibody (abcam, ab205718) for 30 min at room temperature. Signal detection was carried out using a DAB (3,3′-diaminobenzidine) substrate kit, and sections were counterstained with hematoxylin. Slides were dehydrated, cleared, and mounted for microscopic examination using an Olympus microscope at 20 × magnification. Representative images included a scale bar of 50 μm for reference.

For quantitative analysis of immunohistochemical staining, digital images were imported into ImageJ (National Institutes of Health, USA) and analyzed as follows: Images were converted to 8-bit grayscale. Background was subtracted using the “Subtract Background” function (rolling ball radius = 50 pixels). A consistent threshold was applied across all images to isolate DAB-positive staining. Regions of interest (ROIs) covering the tissue area were selected; three random fields per section were analyzed. The “Analyze → Measure” function was used to quantify both the percentage of positive area (positive area/total area × 100%) and the integrated optical density (IOD). The mean value of three fields was calculated for each animal.

### 2.4 RNA extraction, cDNA synthesis, and qPCR analysis

Total RNA was extracted using the RNAeasy™ kit (R0027; Beyotime Biotechnology), following the manufacturer’s protocol. The extracted RNA was reverse transcribed into complementary DNA (cDNA) using SuperMix (R323-01; Vazyme). Quantitative polymerase chain reaction (qPCR) was conducted on the Quantstudio 5 system utilizing SYBR Green PCR Master Mix (Q311-02; Vazyme). *β*-actin served as the internal reference gene for normalization. The primer sequences used were as follows:

β-actin:

Forward: 5’-TTCGCCATGGATGACGATATC-3’

Reverse: 5’-TAGGAGTCCTTCTGACCCATAC-3’

SURF4:

Forward: 5’-ATCATCGCACTTCAGACAATTG-3’

Reverse: 5’-AAACATGCTCTTCCCTTCTGAG-3’

Data were analyzed using the comparative Ct (ΔΔCt) method. Briefly, the threshold cycle (Ct) values of target genes were first normalized to the endogenous reference gene (*β*-actin). The ΔCt value for each sample was calculated as ΔCt = Ct_target − Ct_reference. Subsequently, the ΔΔCt value was determined by comparing each experimental sample to the control group: ΔΔCt = ΔCt_sample − ΔCt_control. Relative gene expression levels were then calculated as 2^−ΔΔCt. All reactions were performed in technical triplicates, and mean values were used for analysis.

### 2.5 Study design and data sources

Our study employed a summary-level two-sample MR framework. Our primary objective was to investigate the causal association between PCOS susceptibility and severity abnormal circulating lipid levels. The Instrumental Variables (IVs) utilized in the analysis were selected in accordance with three fundamental principles (33): (A) robust Associations with HDL, LDL, TG and PCOS; (B) Independence from known confounders; and (C) Influence on solely through exposure factors, excluding alternative pathways.

The data used in this study are entirely sourced from public databases and do not involve any new data collection.

#### 2.5.1 PCOS data

The PCOS GWAS data were obtained from a meta-analysis of seven European cohort studies (34). This meta-analysis of PCOS GWAS boasts a substantial and demographically representative sample size, enhancing its statistical power and generalizability. The PCOS cohort comprised 10,074 individuals diagnosed with PCOS, while the control group consisted of 103,164 healthy female participants (34). After evaluating Cochran’s Q statistic and I^2^, there is no statistically significant heterogeneity between PCOS groups. Different studies have produced similar results, and the genetic effects are consistent across the various diagnostic criteria groups. The data in these databases have been collected with the consent of the original study participants and the related research projects have been approved through their respective ethics review processes. The data have been de-identified to ensure they cannot be traced back to any individuals, thus making them ethically and legally suitable for this research (34). Detailed information is provided in Table 1.

**Table 1:** Data sources for the analysis.

Exposure/outcome	Trait	Consortium/cohort study	Ethnicity	Participates	Pubmed ID
Genetic instruments for polycystic ovarian syndrome	PCOS	The Apollo University of Cambridge Repository	European	10,074	31,805,045
Genetic instruments for lipid-related traits	HDL	UK Biobank		403,943	32,203,549
	LDL			440,546	
	TG			441,016	

#### 2.5.2 Blood lipids data

We obtained the IEU Open GWAS program^1^ for all traits reported in it. This study utilized individual participant data from the UK Biobank (UKBB), which has secured ethical approval from the Research Ethics Committee (REC; approval number: 11/NW/0382) (35). The UKBB has also obtained informed consent from all participants involved. While circulating lipids comprise various components, the clinical features associated with the occurrence of PCOS metabolic syndrome often manifest as elevated TG, decreased HDL, and increased LDL. Therefore, our study selected HDL, LDL, and TG to indicate lipid abnormalities. We utilized genetic variants from GWAS involving both genders to instrument circulating lipids, as initial analyses indicated that sex-specific instruments did not significantly alter the outcomes. We exported GWAS summary-level data pertaining to HDL, LDL, and TG, with the following numbers of observations: LDL (*n* = 403,943), HDL (*n* = 440,546), and TG (*n* = 441,016) (35). Detailed information is presented in Table 1.

#### 2.5.3 Genetic instrument selection

The selection of IVs is particularly important for the success of MR study design because genetic variants are commonly used as representative exposure factors in MR design (36). From the IEU database, we selected genetic variants significantly associated with HDL, LDL, and TG for a genome-wide significance analysis (*p* < 5 × 10^−8^). For the genome-wide significance analysis within the context of PCOS, we observed that when we set the significance threshold at a *p* < 5 × 10^−8^, only one SNP met this stringent criterion. Therefore, to ensure a more robust analysis, we chose to adjust the significance threshold to *p* < 5 × 10^−6^ for our study. This adjustment allowed for a more comprehensive assessment of genetic variants associated with the condition under investigation, given the limited number of variants meeting the initial stringent threshold. We performed chain disequilibrium analysis to ensure independence between SNPs, where SNPs with r^2^> 0.001 were removed. In addition, the F statistic (*F* > 10) was used to ensure the inclusion of robust IVs while excluding weaker IVs. Full details of the selected SNPs are available in the Supplementary Figures S1–S8.

#### 2.5.4 Statistical analyses for MR study

Inverse Variance Weighting (IVW) method was employed as our primary statistical analysis. Secondary methods included MR-Egger, weighted median, simple mode, weighted mode, and MR-PRESSO. MR-Egger utilizes weighted regression to estimate causal effects, even when instrumental variable assumptions are invalid. The weighted median method assumes at least 50% of genetic instruments are valid for reliable association estimates.

In the IVW analysis, we assessed SNP heterogeneity with *p* < 0.05 and a = 0.05 as significance thresholds. To examine horizontal pleiotropy between genetic variants and confounders, we conducted the MR-Egger test (Supplementary Figures S2, S6). For potential bias assessment, we performed leave-one-out analysis to evaluate individual SNP influence on MR findings (Supplementary Figures S4, S8). Furthermore, we validated results using the MR-PRESSO method, which detects outliers, generates corrected estimates, and assesses significance distortion. The simple and weighted modes were employed to assess the association. In multivariate MR analysis, IVW served as the primary statistical method.

During the analysis, we queried the PhenoScanner database^2^ to identify instrumental variables (IVs) associated with other phenotypes that could potentially confound the outcomes. IVs including rs11688682, rs10773000, rs12986742, rs1408579, rs116843064, rs13389219, rs1800961, rs247975, rs62033400, rs2613499, rs6857, rs7609422, rs3768321, rs429358, rs4686392, rs62271373, rs72964564, rs11563251, rs8074454, rs4755720, rs10513801, rs3732359, rs17619973, rs1225053, rs3814883, rs1431659, rs921971, and rs2965169 which were significantly associated with hyperandrogenism, IR, type 2 diabetes mellitus, body fat percentage, BMI, and age at menarche. These may act as confounding variables in the PCOS phenotype. To address this concern, we excluded these IVs from the analysis and reevaluated the MR analysis. This process resulted in consistent conclusions with the initial analysis.

We assessed odds ratios (OR) and 95% confidence intervals (CI), along with two-tailed *p*-values. In the IVW model, *p* < 0.05 was deemed statistically significant, and this association direction remained consistent across the other five models (MR-Egger’s method, weighted median, simple mode, weighted mode, and MR-PRESSO method). All analyses were conducted using R (version 4.2.1, Vienna, Austria) with the ‘Two Sample MR’ package (version 0.5.6, Bristol, UK), ‘MVMR’ package (version 0.3, Bristol, UK), and ‘MR-PRESSO’ package (version 1.0, New York, NY, USA). Data were visualized and analyzed using STATA 12.0 and R software.

### 2.6 Statistical analysis

Data analysis was performed using Prism 9.0. Results from a minimum of three independent experiments are presented as mean ± standard deviation. Normality was assessed using the Shapiro–Wilk test. For normally distributed data, group differences were evaluated using one-way ANOVA, followed by Tukey’s HSD or Games-Howell post-hoc tests depending on variance homogeneity. For non-normally distributed data, the Kruskal–Wallis test was used, followed by Dunn’s test with Bonferroni correction for multiple comparisons. For comparisons between two groups, the Student’s t-test was applied when data were normally distributed. A *p*-value < 0.05 was considered statistically significant.

## 3 Results

### 3.1 PCOS may lead to lipid disorders rather than vice versa

#### 3.1.1 Univariable MR analysis and multivariable MR analysis: circulating lipids and PCOS

**Figure 1:**
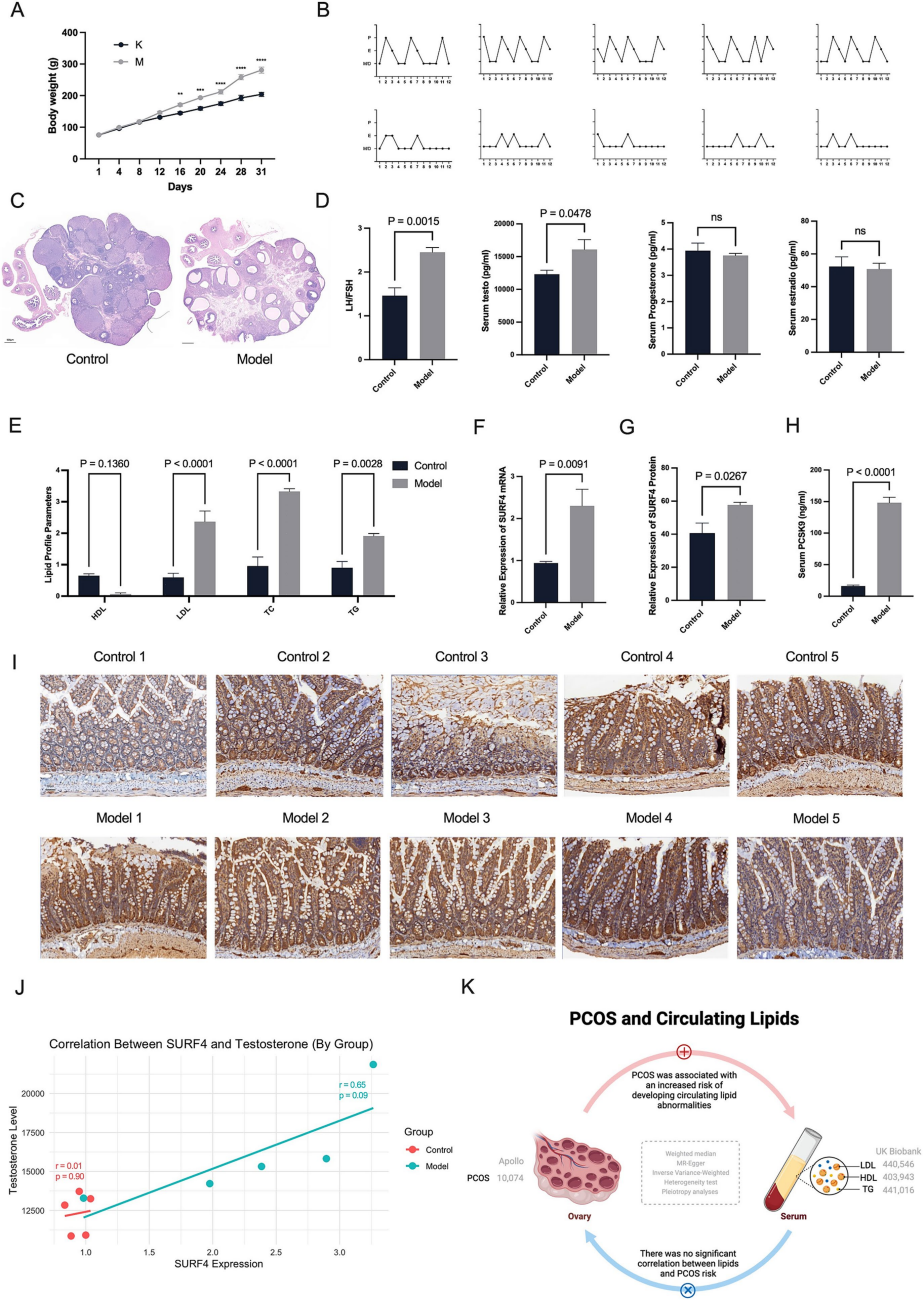
Hyperandrogenism may drive the effects of intestinal SURF4 on lipid metabolism in PCOS. **(A)** Body weights of rats were recorded daily (*n* = 5 per group). **(B)** Estrous cycles were monitored 14 days after treatment (*n* = 5). *X*-axis: Days; *Y*-axis: P (Proestrus, follicular development), E (Estrus, ovulation and receptivity), M (Metestrus, corpus luteum formation), D (Diestrus, progesterone dominance/regression). **(C)** Representative ovarian histology images stained with hematoxylin and eosin (H&E) (scale bar = 500 μm). **(D,E,H)** Serum levels of LH, FSH, testosterone, PCSK9, and lipid profiles (TG, TC, HDL-C, LDL-C) were measured using commercial ELISA kits. **(F,G,I)** Intestinal SURF4 expression was assessed at both mRNA and protein levels using qPCR and immunohistochemistry, respectively. **(J)** Pearson correlation analysis showed a strong positive correlation between SURF4 expression and serum testosterone levels in the Model group (*r* = 0.809, *p* = 0.097), while no significant correlation was observed in the Control group (*r* = 0.101, *p* = 0.872). Data are presented as mean ± standard error of the mean (SEM). *p < 0.05, **p < 0.01, ****p < 0.0001.

The association between the three circulating lipid traits and PCOS in the IEU and Apollo database populations are shown in Figure 1K.

**Figure 2:**
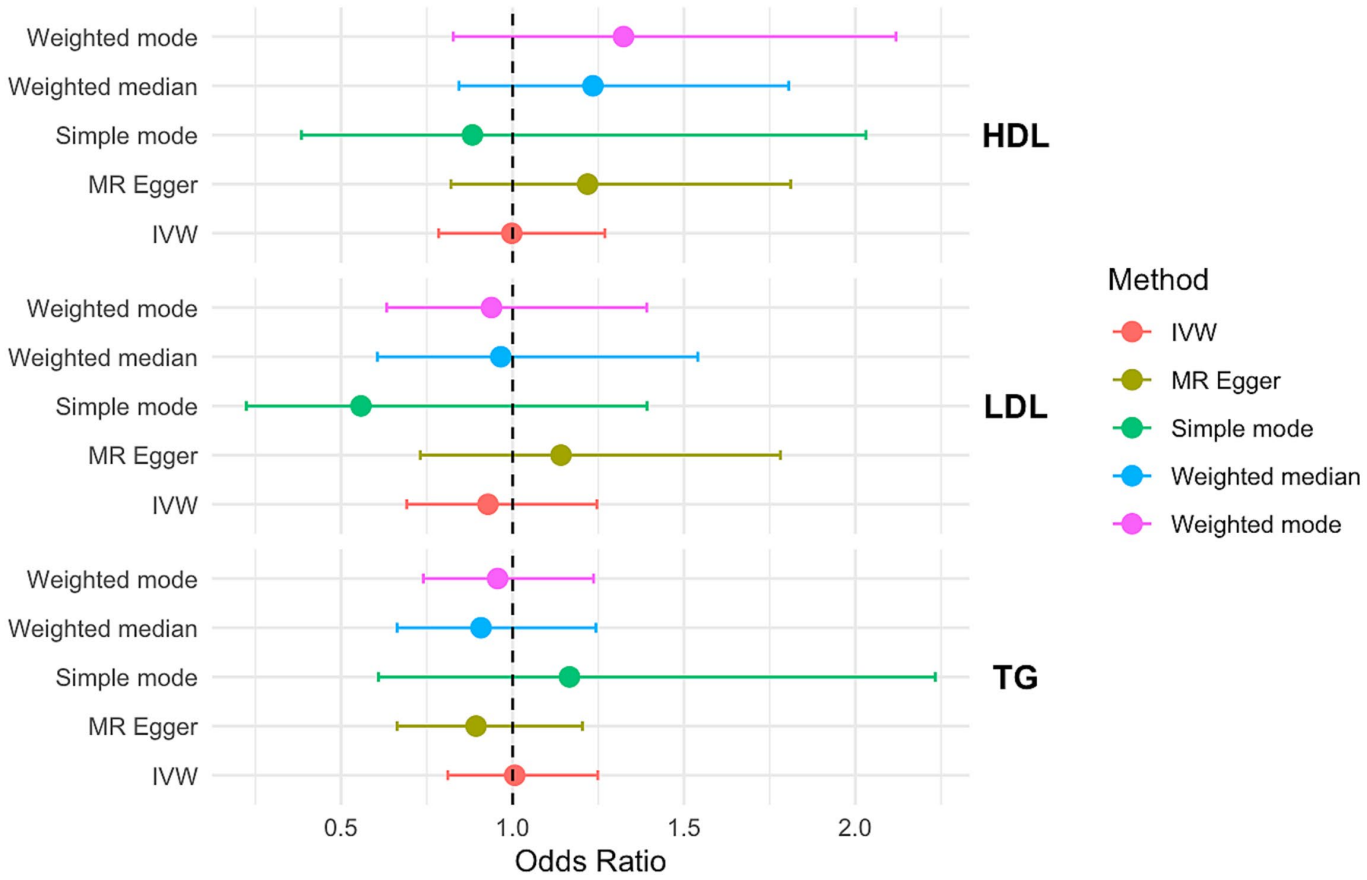
OR and 95% CI of the causal relationship between circulating lipid traits and PCOS. Primary analyses used the inverse-variance weighted (IVW) method. Sensitivity analyses included MR-Egger regression and weighted median approaches. Heterogeneity was assessed using Cochran’s Q test, and horizontal pleiotropy was evaluated with the MR-Egger intercept.

In the IVW MR analysis, the ORs and corresponding 95% CIs for HDL, LDL, and TG indicated that none of them were significantly associated with PCOS risk (Figure 2). Consistent results were obtained from MR-PRESSO analysis. Furthermore, the MR-Egger intercept values of 0.198, 0.132, −0.112 (Supplementary Table S11) suggested no directional pleiotropy in the genetic variation.

**Figure 3:**
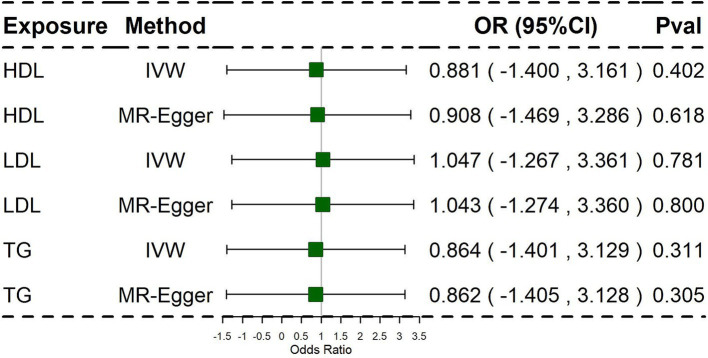
Associations of lipid traits in multivariable MR using the Inverse Variance Weighted method and MR-Egger method. Primary analyses used the inverse-variance weighted (IVW) method. Sensitivity analyses included MR-Egger regression.

To further address potential bias in assessing the impact of HDL, LDL, and TG on PCOS, we conducted Multivariable-Mendelian Randomization (MVMR) analysis. The results of the multivariate MR analysis are presented in Figure 3.

In this analysis, we used IVW as the primary analysis method. The findings of the multivariate MR analysis revealed that HDL, LDL, and TG were not significantly associated with PCOS. Similarly, the multivariate MR analysis did not suggest that potential pleiotropy reduced instrument validity (Supplementary Table S12).

#### 3.1.2 Univariable MR analysis: PCOS and circulating lipids

In clinical observations, women with PCOS often exhibit a combination of circulating lipid and serum lipid metabolism abnormalities (19). The potential impact of dyslipidaemia on the progression of PCOS has been suggested in clinical recommendations for clinicians. Therefore, we proceeded to investigate whether PCOS is a key factor in the alterations of circulating lipid levels.

**Figure 4:**
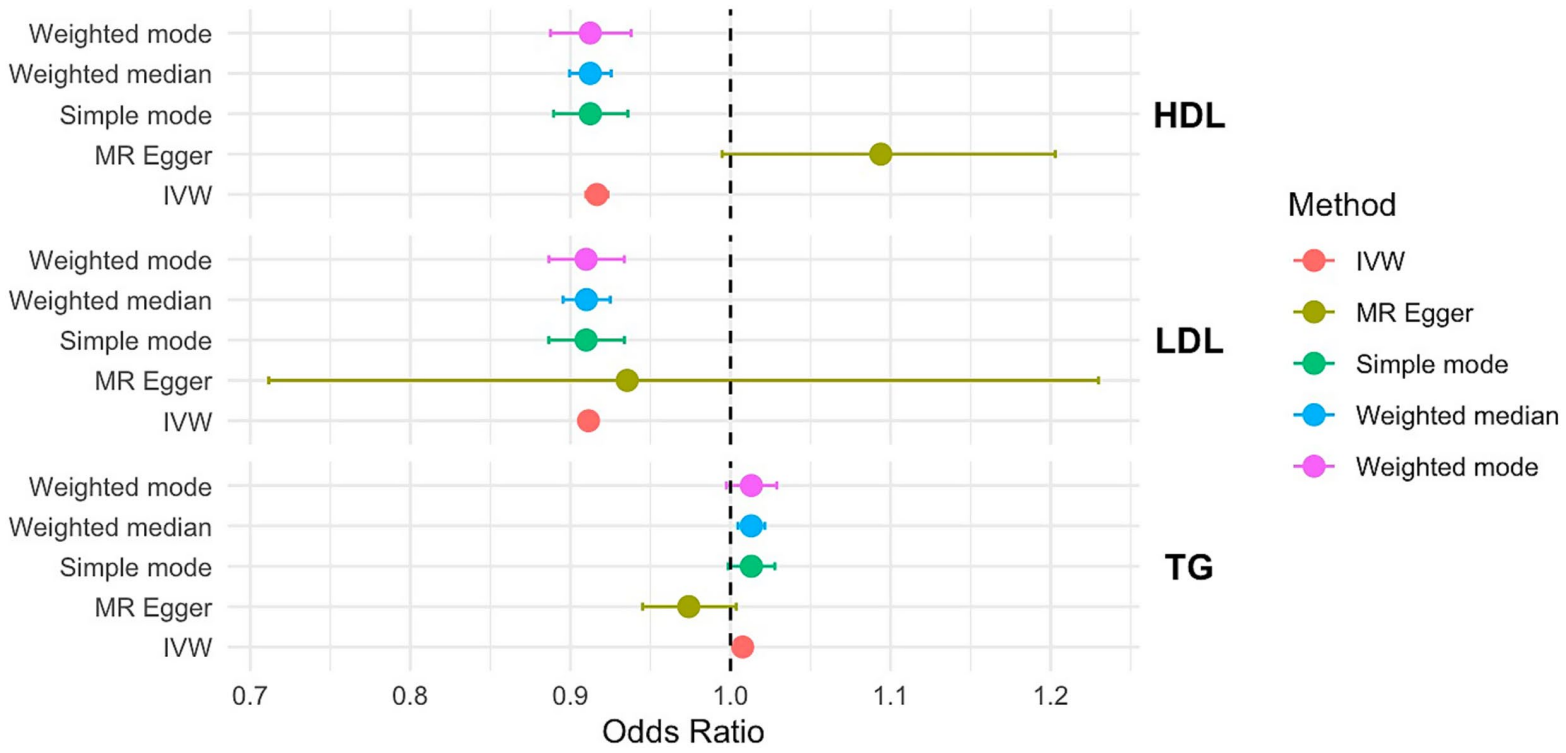
OR and 95% CI of the causal relationship between the risk of PCOS and circulating lipid traits. Primary analyses used the inverse-variance weighted (IVW) method. Sensitivity analyses included MR-Egger regression and weighted median approaches. Heterogeneity was assessed using Cochran’s Q test, and horizontal pleiotropy was evaluated with the MR-Egger intercept.

The relationship between HDL, LDL, TG, and PCOS is shown in Figure 4.

In the IVW MR analysis, HDL, LDL, TG were significantly associated with PCOS risk (*p* < 0.001, HDL: OR=0.916, 95%CI=0.909–0.923; LDL: OR=0.911, 95%CI=0.910–0.913; TG: OR=1.000, 95%CI=1.00–1.012). Consistent results were obtained from the MR-PRESSO analysis. The IVW analysis revealed no evidence of potential hybrid advantage. Additionally, the MR-Egger intercepts were 0.09, −0.07, and −0.03, indicating no directional pleiotropy in the genetic variation (Supplementary Table S13).

### 3.2 Hyperandrogenaemia and circulating lipid abnormalities in rats with PCOS

Since the Mendelian randomization analyses suggest that PCOS drives serum lipid abnormalities rather than lipid abnormalities predisposing individuals to PCOS. Here, we explored the possible causes of PCOS-induced dyslipidaemia.

We successfully established a rat model of PCOS induced by letrozole gavage combined with a high-fat diet (HFD). The model rats showed marked metabolic disturbances, including weight gain, from day 12 onwards (Figure 1A). In addition, disturbances in the oestrous cycle were observed in the model group, accompanied by polycystic ovarian changes (Figures 1B,C). At the end of the modelling period, PCOS-like serological alterations were confirmed by elevated luteinizing hormone (LH) to follicle-stimulating hormone (FSH) ratios (*p* = 0.0129) and increased testosterone levels (*p* = 0.0390) (Figure 1D). Lipid analysis further indicated significant dysregulation of lipid metabolism in the model rats (Figure 1E). Serum analyses of rats with PCOS revealed low HDL, high LDL, high TC, and high TG levels.

### 3.3 Elevated intestinal SURF4 and serum PCSK9 levels in rats with PCOS

SURF4 is a critical protein involved in intestinal lipid absorption and transport. Studies on intestinal SURF4 knockout mice have shown lipid accumulation in the intestinal lumen, insufficient serum lipid levels, and premature mortality within 2 days of birth (37). These findings highlight the pivotal role of intestinal SURF4 in lipid absorption. Previous studies have also demonstrated sex-specific differences in the regulation of intestinal SURF4 expression under HFD conditions in inducible heterozygous mice, with distinct responses observed in both male and female mice (31).

We employed two well-established PCOS models, the letrozole + HFD model primarily reflects the metabolic and endogenous androgen elevation characteristics of PCOS, whereas the DHEA model represents exogenous androgen-induced PCOS.

To investigate whether testosterone elevation in female mice influences intestinal SURF4 functionality, we measured intestinal SURF4 protein and mRNA levels (Figures 1F,G,I). Our results showed a significant upregulation of intestinal SURF4 in rats with PCOS compared to the control group.

Given the established positive correlation between elevated serum PCSK9 levels, SURF4 upregulation, and hyperlipidemia—particularly through SURF4-mediated hepatic lipoprotein packaging and subsequent metabolic inactivation—we measured serum PCSK9 levels. Rats with PCOS exhibited markedly elevated serum PCSK9 levels (Figure 1H).

We conducted correlation analyses between intestinal SURF4 and PCSK9, serum lipid profiles, and androgen levels (Figure 1J). In the disease state (model group), the correlation between SURF4 and testosterone levels suggested a link between elevated SURF4 expression and androgenic alterations associated with PCOS. However, in the normal state (control group), this relationship may have been masked by regulatory mechanisms.

Hormonal changes are crucial factors in the induction of SURF4 by a high-fat diet. Exogenous testosterone, to some extent, promotes the elevation of SURF4 in females. The residual levels of Surf4 protein in female intestinal Surf4-IKO mice are higher than those in male Surf4-IKO mice, indicating the hormone-specific regulation of intestinal Surf4 (32). To further elucidate the role of androgen regulation on intestinal Surf4 and serum PCSK9 expression in PCOS rats, we established a subcutaneous DHEA injection-induced PCOS rat model. This is another classic method of inducing PCOS, commonly used to simulate hyperandrogenemia, particularly focusing on gender-specific characteristics and the effects of androgen levels. Our study found that the body weight of the DHEA-induced model was slightly higher than that of the blank control group, although the difference was not statistically significant (*p* > 0.05). However, the DHEA rat model also exhibited disrupted estrous cycles, polycystic ovary-like changes, an elevated LH/FSH ratio (*p* < 0.01), a significant increase in testosterone (*p* < 0.01), and elevated levels of LDL, TC, and TG (Supplementary Figure S9). This indicates that DHEA administration successfully induces ovarian polycystic changes in rats. Further investigation revealed that both the mRNA and protein levels of intestinal SURF4 were elevated in the DHEA-induced PCOS rats, suggesting that androgen elevation, whether endogenous or exogenous, impacts the expression of intestinal SURF4 in PCOS rats. These findings suggest that hyperandrogenism, a hallmark of PCOS, maybe a factor driving the influence of intestinal SURF4 on lipid metabolism.

## 4 Discussion

Our findings indicate that the characteristic hyperandrogenism and metabolic dysfunction in PCOS may lead to elevated lipid levels through the upregulation of intestinal SURF4, underscoring its potential as a regulatory factor in the pathogenesis of PCOS-associated dyslipidaemia.

There is a clinical consensus that dyslipidaemia is closely associated with the development of PCOS. The prevalence of dyslipidaemia among patients with PCOS is reported to be 52.96%, which is approximately twice as high as that in the control group (38). This abnormality affects the metabolic and reproductive function of women by influencing the hormonal environment of the ovaries, steroid synthesis (39), and endometrial tolerance (40). Studies have reported a variety of conclusions regarding the relationships between LDL, HDL, and TG levels and endocrine metabolism in women. Women with PCOS who maintain a normal BMI still show higher lipid levels than the general population, suggesting that dyslipidaemia may be associated with an increased risk of PCOS, independent of body weight and BMI (41, 42). Additionally, studies conducted in different populations have revealed diverse profiles of dyslipidaemia, with elevated LDL levels associated with PCOS in white women, whereas low HDL levels are most common among Chinese patients (9, 21). These studies have demonstrated a significant linear association between lipids, body fat percentage, total body fat mass and PCOS (43, 44). Using univariate and multivariate MR analyses, we examined the causal relationships between genetic lipid traits and PCOS risk. These findings indicate that PCOS leads to serum lipid abnormalities rather than vice versa. Our findings suggest that PCOS may contribute to serum lipid abnormalities, though causality requires further validation across diverse populations. In addition, the relatively limited number of SNPs used as instrumental variables and potential population heterogeneity may have reduced statistical power, thereby contributing to the wide confidence intervals observed. To strengthen these findings, we further validated the MR results using animal models.

Recent studies have demonstrated that an HFD does not induce elevated intestinal SURF4 levels in female SURF4 knockout mice. Compared to wild-type mice, female SURF4 knockout mice exhibit partial resistance to HFD, characterized by reduced intestinal lipid absorption and secretion and decreased serum lipid levels. In contrast, male SURF4 knockout mice show increased intestinal SURF4 expression under HFD conditions, with lipid metabolism alterations similar to those observed in wild-type mice (32). Elevated androgen levels, a hallmark of PCOS, may play a critical role in the regulation of intestinal SURF4 expression and subsequent lipid metabolic disturbances. Similarly, our study demonstrated a sex-specific pattern of intestinal SURF4 expression, showing varying degrees of upregulation in female PCOS rats with hyperandrogenism induced by both endogenous and exogenous sources.

Based on previous studies and our current findings, we propose that intestinal SURF4 plays a critical role in female-specific metabolic disorders, particularly in PCOS and its associated hyperandrogenic state. Our study demonstrates that intestinal SURF4 expression is regulated by sex and androgen levels, with a significant increase observed in hyperandrogenic female rats. These results suggest that targeting intestinal SURF4 may offer a therapeutic avenue for improving PCOS-associated dyslipidaemia. The observed sex-specific effects of intestinal SURF4 on lipid metabolism further underscore its potential role as a key node linking androgen signaling to metabolic regulation. Future studies could explore androgen receptor-mediated regulation of SURF4 transcription to elucidate sex-specific mechanisms.

## 5 Strengths and limitations

This study used univariable and multivariable Mendelian randomization (MR) to explore the causal relationship between circulating lipids and PCOS, enhancing result reliability and reducing biases. MR mitigates reverse causality and confounding factors, while leveraging the IEU Open GWAS database strengthens genetic associations. By cross-validating animal models with human genetic data, we gain a comprehensive understanding of lipid metabolism in PCOS. This study focuses on the potential mechanisms underlying lipid metabolic disturbances in PCOS, identifying intestinal SURF4 as a potential hormone-related protein. By detecting the elevated levels of intestinal SURF4 and serum PCSK9 in PCOS rats, the study offers valuable insights into how androgen excess may impact lipid metabolism. Moreover, the research underscores the importance of sex-specific differences in lipid metabolism by investigating the role of elevated androgens in regulating intestinal SURF4 expression and lipid metabolism.

However, MR provides overall effects rather than direct mechanisms. While controlling for known confounders like type 2 diabetes and obesity, unexamined factors may still influence results. Our sample was mostly European, limiting generalizability to other ethnic groups. Future research should include more diverse datasets to better understand lipid-PCOS links and identify broader interventions. Because of current experimental constraints, we were unable to establish gene knockout animal models or further explore primary intestinal cells from female mice to evaluate the precise role of androgens in the regulation of intestinal lipid absorption and metabolism. Future research should focus on elucidating the molecular mechanisms linking hyperandrogenism, intestinal SURF4, and lipid metabolism using advanced *in vivo* and *in vitro* models.

## 6 Conclusion

Our study provides novel insights into the role of PCOS in dyslipidaemia, with intestinal SURF4 potentially playing a key regulatory role in lipid metabolism. The upregulation of intestinal SURF4 in PCOS and its potential regulation by elevated androgen levels highlights a significant target for addressing lipid metabolic disorders associated with PCOS. MR analyses suggested that PCOS drives serum lipid abnormalities rather than lipid abnormalities, causing a predisposition to PCOS. These findings provide valuable insights into potential avenues for therapeutic intervention and emphasize the importance of PCOS as a risk factor for dyslipidaemia in clinical practice.

## Data Availability

The datasets presented in this study can be found in online repositories. The names of the repository/repositories and accession number(s) can be found in the article/Supplementary material.

## Footnotes

^1^ https://gwas.mrcieu.ac.uk/

^2^ http://www.phenoscanner.medschl.cam.ac.uk/
