# Supplementary material for: Intestinal SURF4 in dyslipidaemia and female-specific metabolic disorders: insights from rats with polycystic ovary syndrome
Source: Front Nutr. 2025 Sep 17;12:1644496. doi: 10.3389/fnut.2025.1644496 (PMC12483925; doi:10.3389/fnut.2025.1644496)

Supplementary Figure S1-S9 Legends

Figure S1. Scatter plot of MR effect size for causal associations between lipid traits and PCOS

Figure S2. Forest plot of MR effect size using MR-Egger and IVW methods for causal associations between lipid traits and PCOS.

Figure S3. Funnel plot of causal associations between lipid traits and PCOS.

Figure S4. Leave-one-out plot to assess if a single variant is driving the associations between lipid traits and PCOS.

Figure S5. Scatter plot of MR effect size for causal associations between PCOS and lipid traits.

Figure S6. Forest plot of MR effect size using MR-Egger and IVW methods for causal associations between PCOS and lipid traits.

Figure S7. Funnel plot of causal associations between PCOS and lipid traits.

Figure S8. Leave-one-out plot to assess if a single variant is driving the associations between PCOS and lipid traits.

Figure S9

(A) Body weights of rats were recorded daily (n = 5 per group).

(B) Estrous cycles were monitored 14 days after treatment (n = 5). X-axis: Days; Y-axis: P (Proestrus, follicular development), E (Estrus, ovulation and receptivity), M (Metestrus, corpus luteum formation), D (Diestrus, progesterone dominance/regression).

(C) Representative ovarian histology images stained with hematoxylin and eosin (H&E) (scale bar = 500 μm).

(D, E, H) Serum levels of LH, FSH, testosterone, PCSK9, and lipid profiles (TG, TC, HDL-C, LDL-C) were measured using commercial ELISA kits.

(F, G) Intestinal SURF4 expression was assessed at both mRNA and protein levels using qPCR and immunohistochemistry, respectively.

(I) Pearson correlation analysis revealed a strong positive correlation between intestinal SURF4 expression and serum testosterone levels in the DHEA group (r = 0.884, p = 0.047), whereas no significant correlation was found in the Control group (r = 0.417, p = 0.485).

Data are presented as mean ± standard error of the mean (SEM). *p < 0.05, **p < 0.01, ****p < 0.0001.

Figure S1. Scatter plot of MR effect size for causal associations between lipid traits and PCOS

A: HDL and PCOS


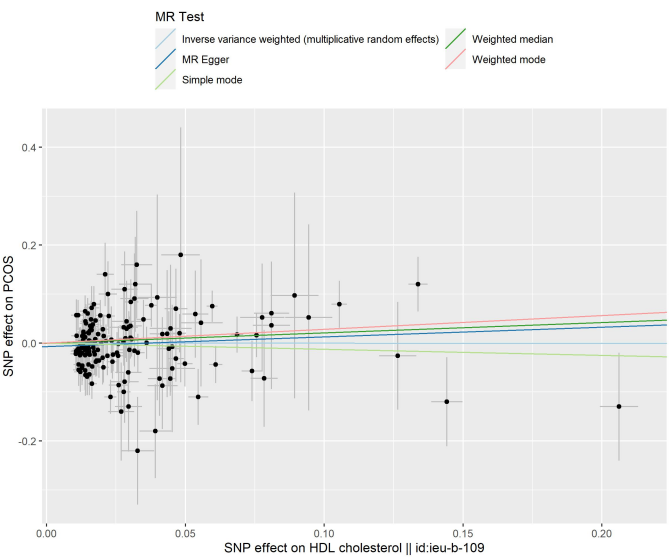


B: LDL and PCOS


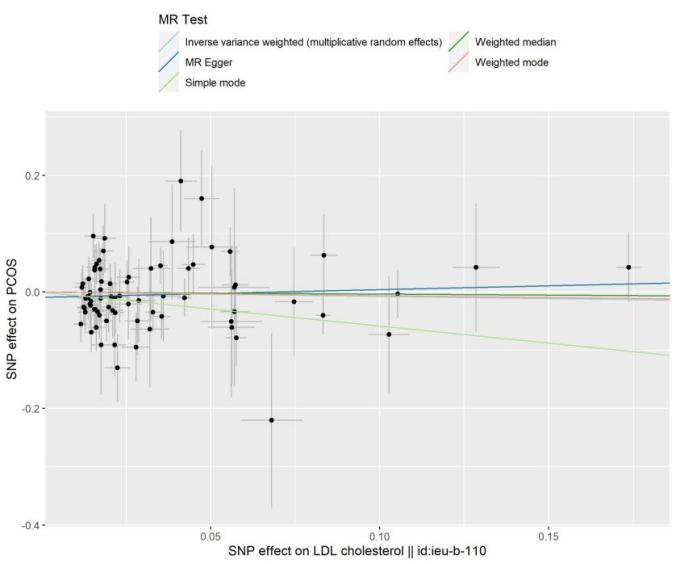


C: TG and PCOS


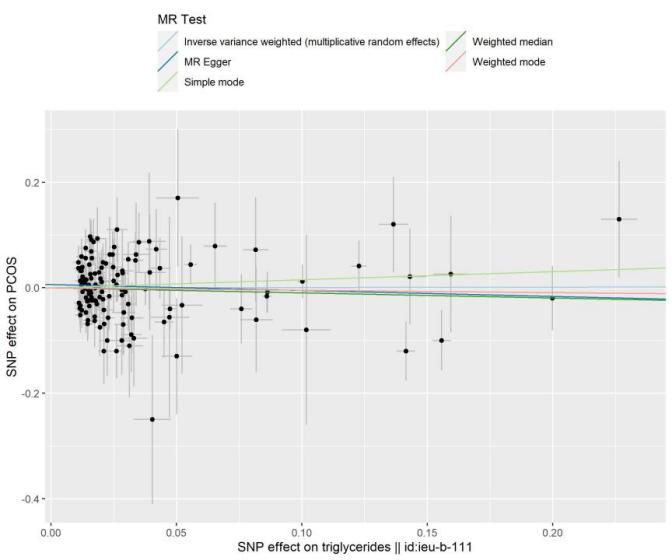


Figure S2. Forest plot of MR effect size using MR-Egger and IVW methods for causal associations between lipid traits and PCOS.

A: HDL and PCOS


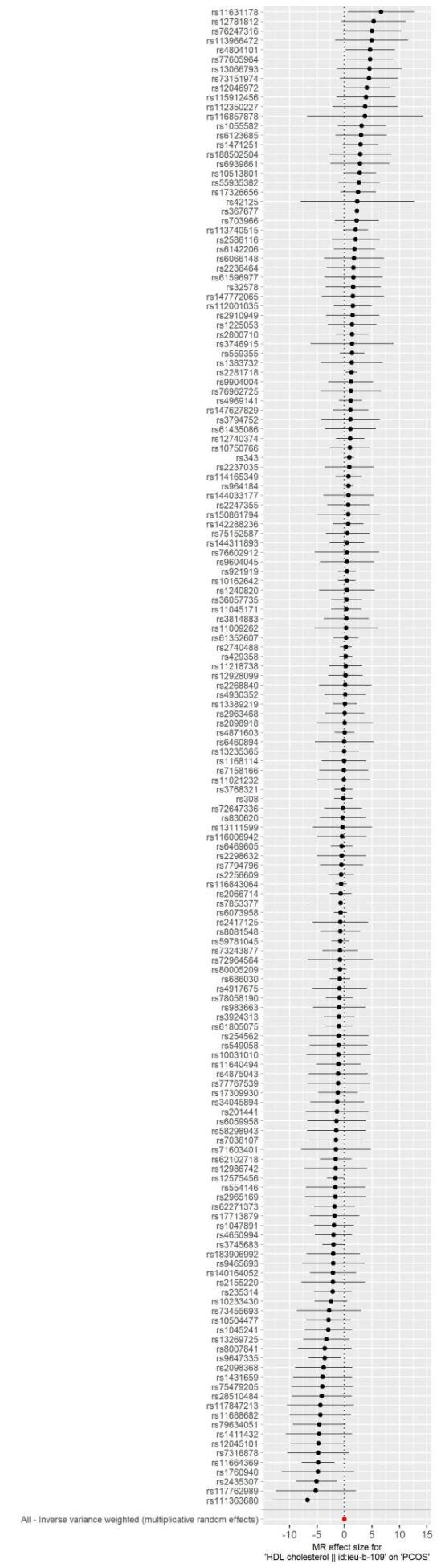


B: LDL and PCOS


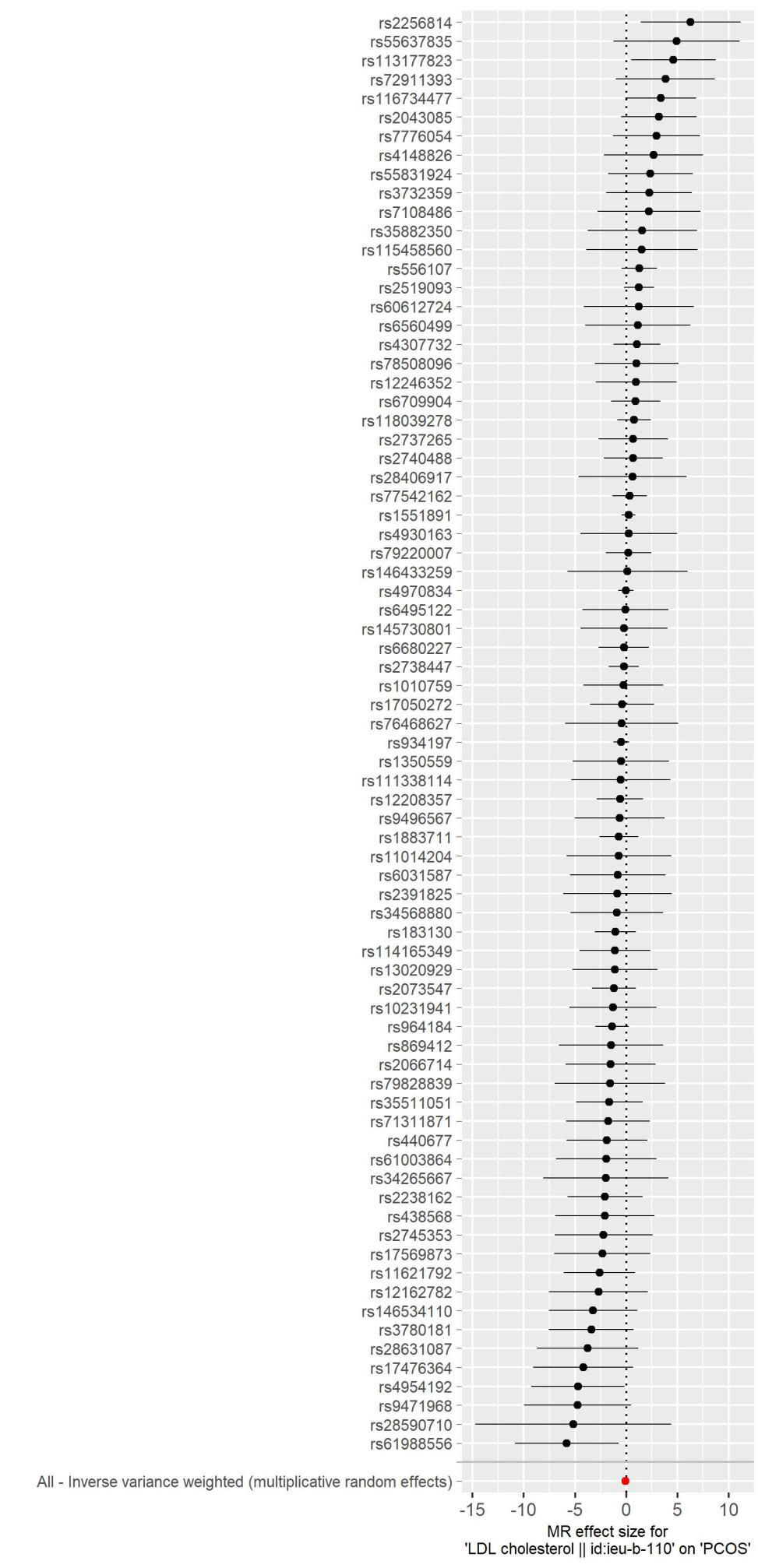


C: TG and PCOS


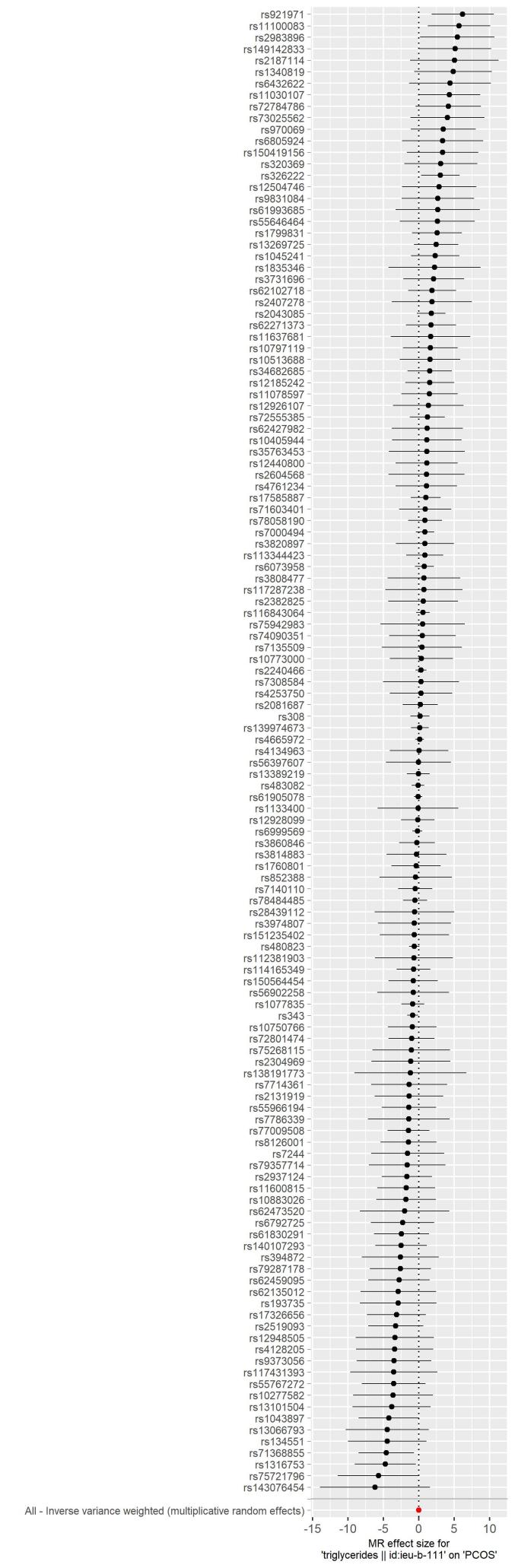


Figure S3. Funnel plot of causal associations between lipid traits and PCOS.

A: HDL and PCOS


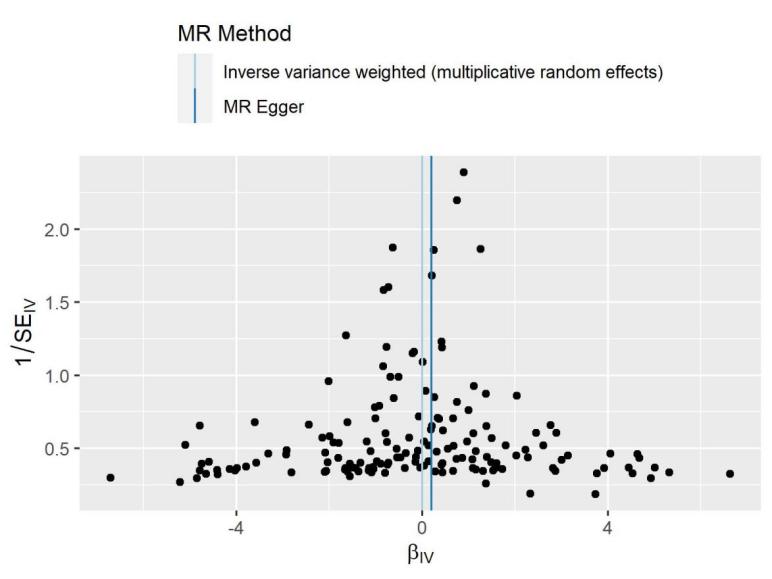


B: LDL and PCOS


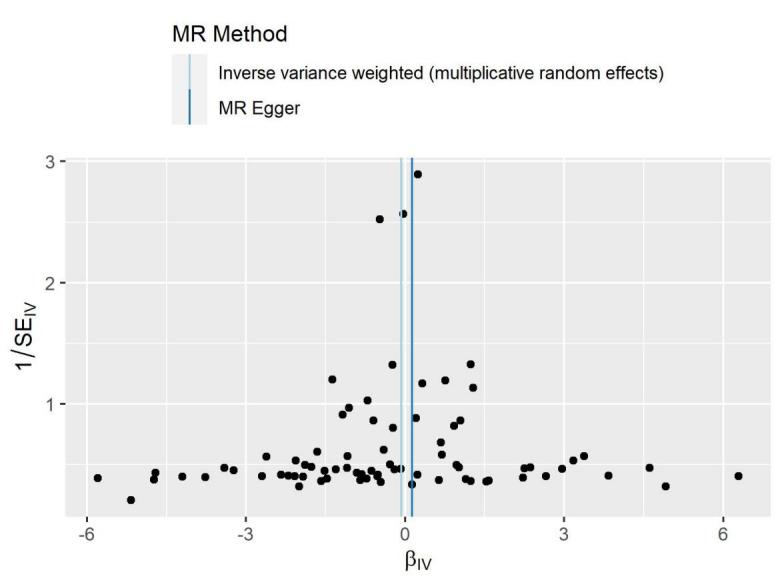


C: TG and PCOS


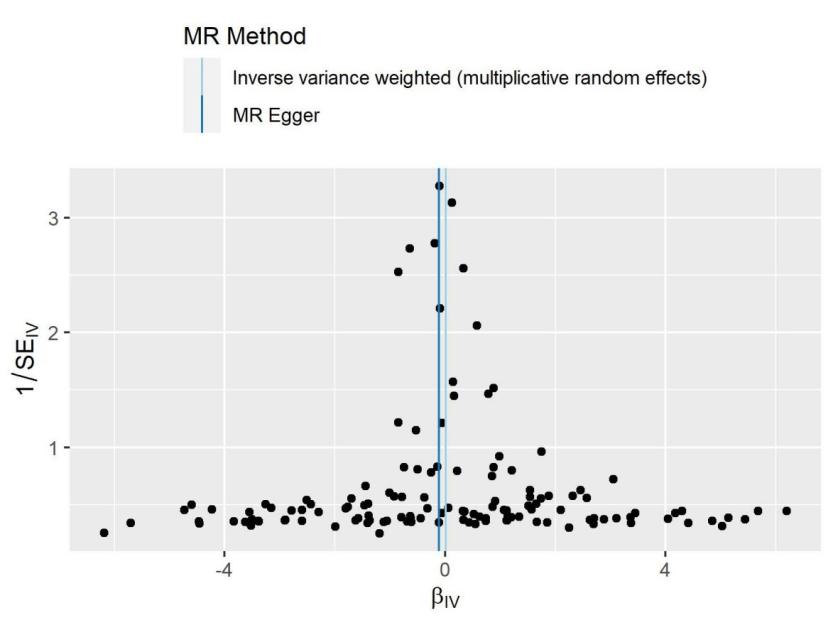


Figure S4. Leave-one-out plot to assess if a single variant is driving the associations between lipid traits and PCOS.

A: HDL and PCOS


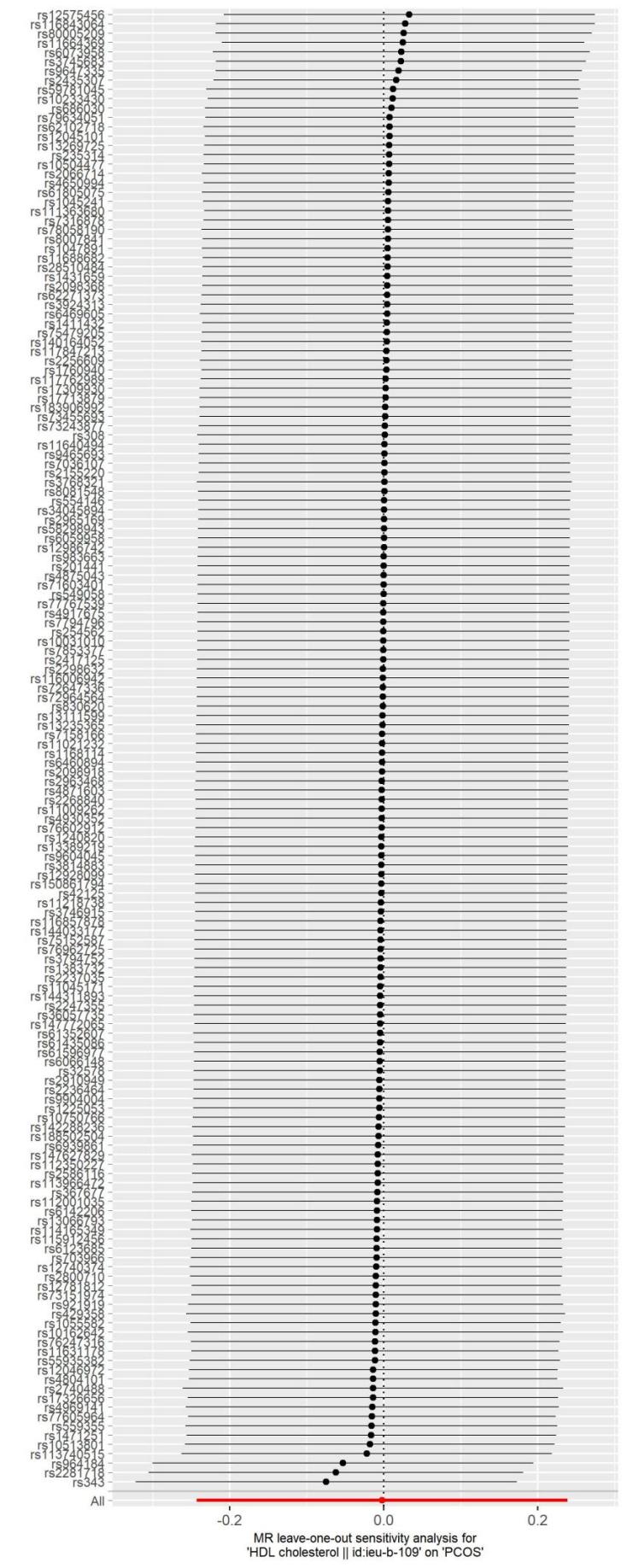


B: LDL and PCOS


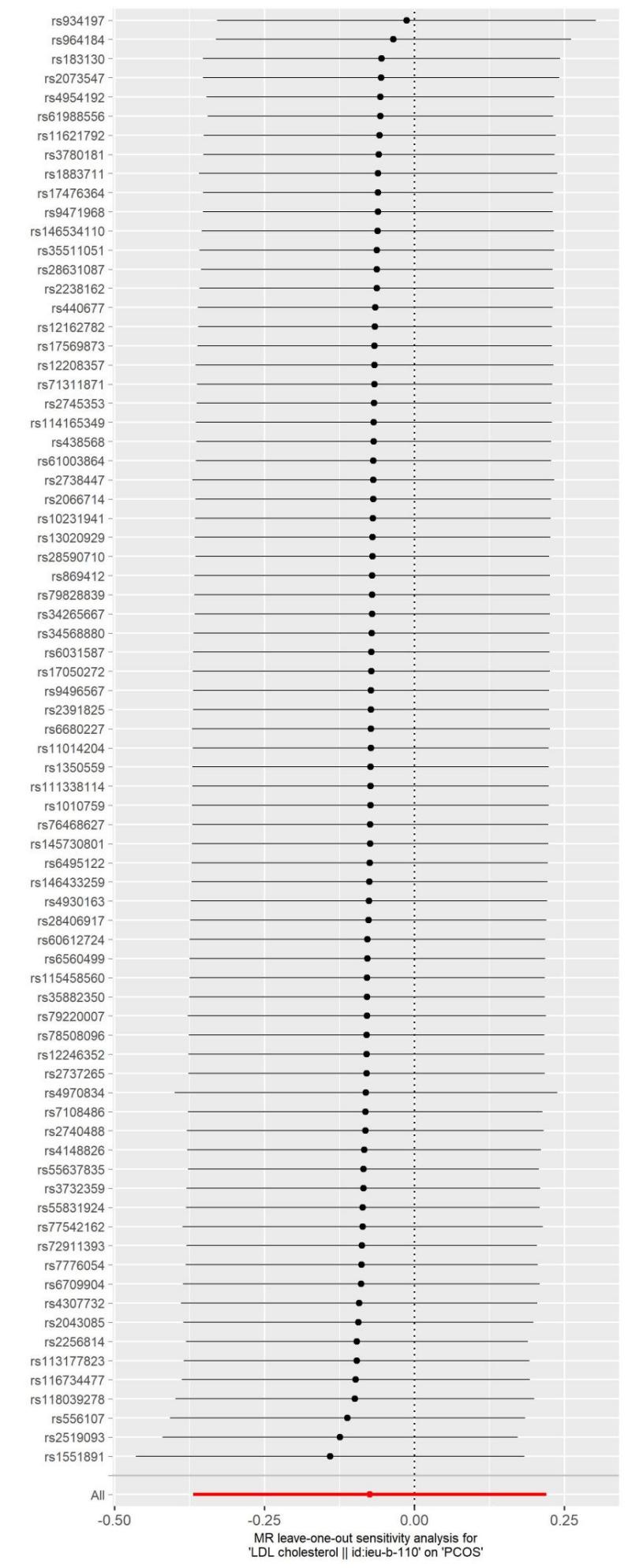


C: TG and PCOS


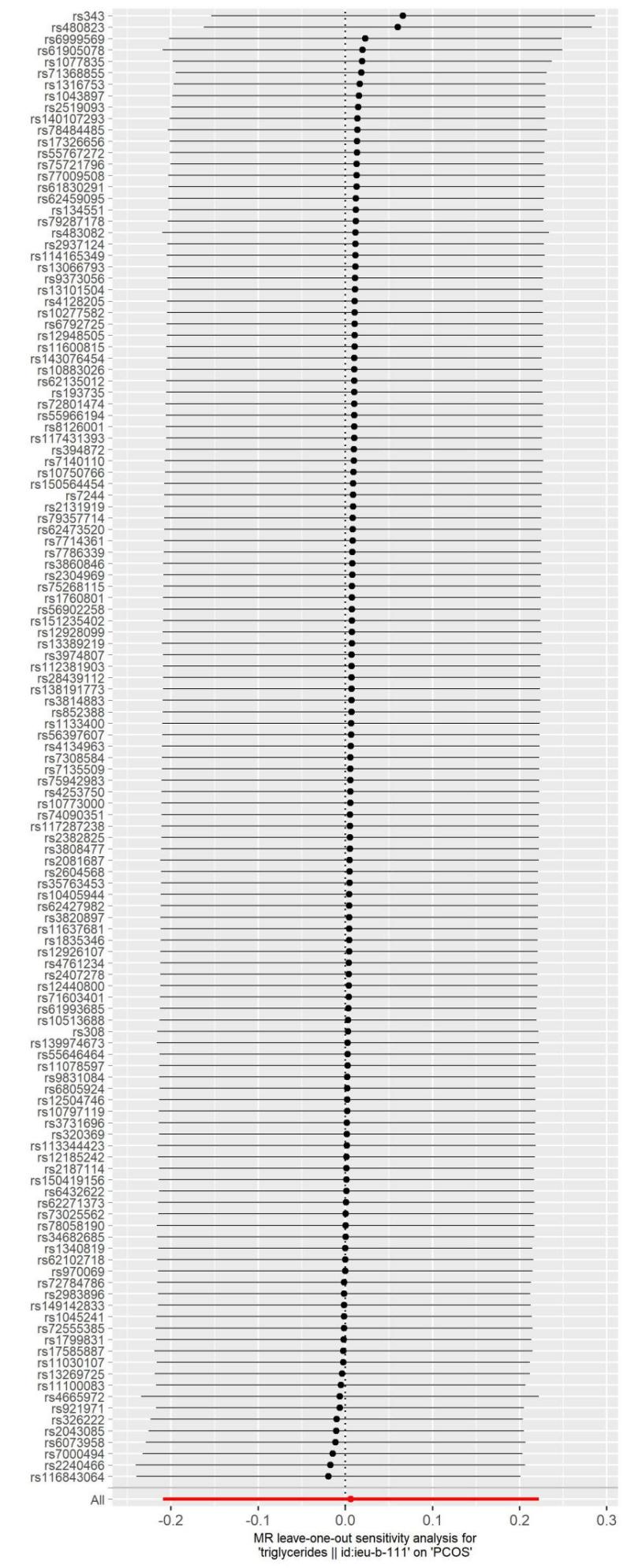


Figure S5. Scatter plot of MR effect size for causal associations between PCOS and lipid traits.

A: HDL and PCOS


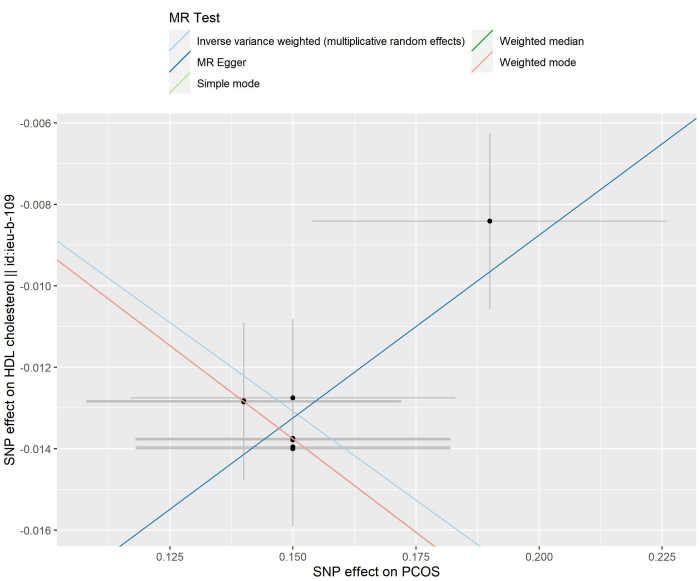


B: LDL and PCOS


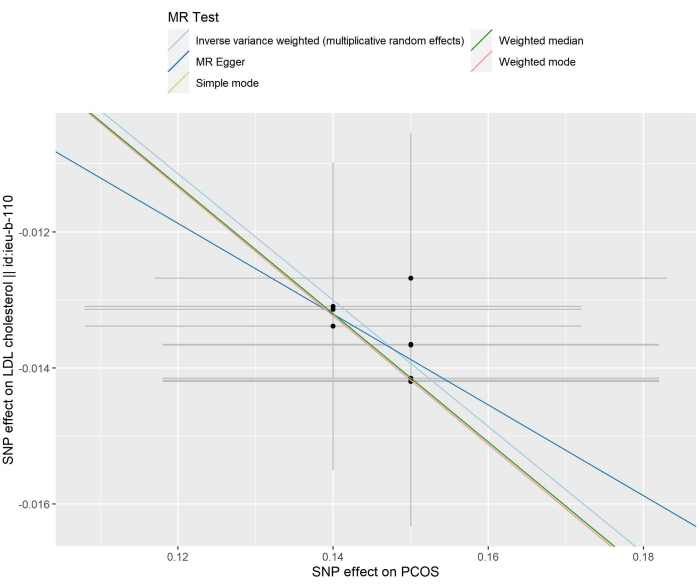


C: TG and PCOS


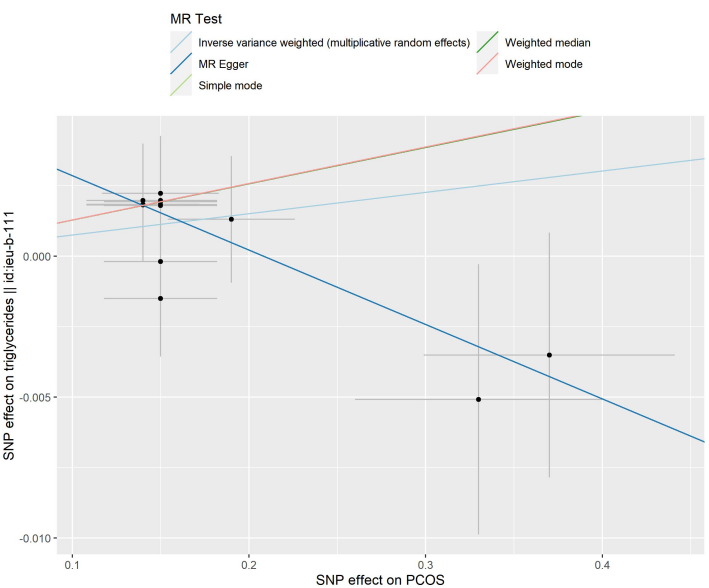


Figure S6.Forest plot of MR effect size using MR-Egger and IVW methods for causal associations between PCOS and lipid traits.

A: HDL and PCOS


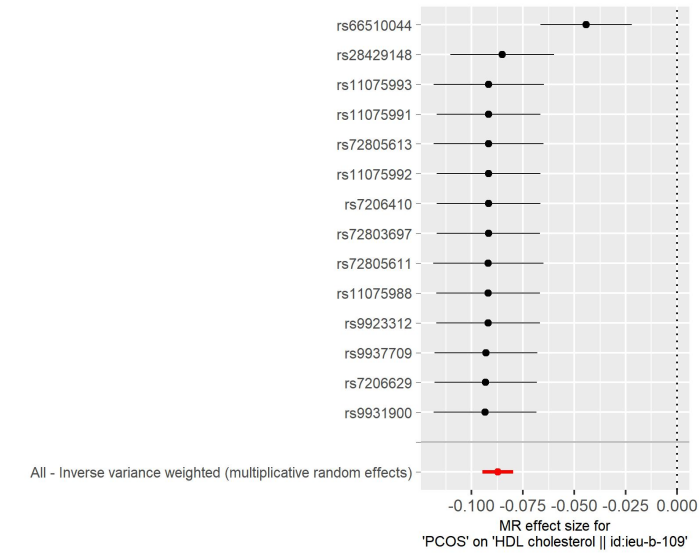


B: LDL and PCOS


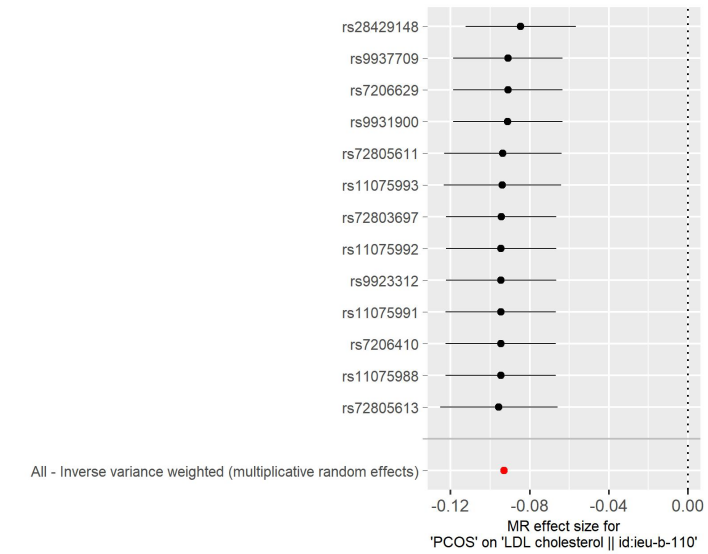


C: TG and PCOS


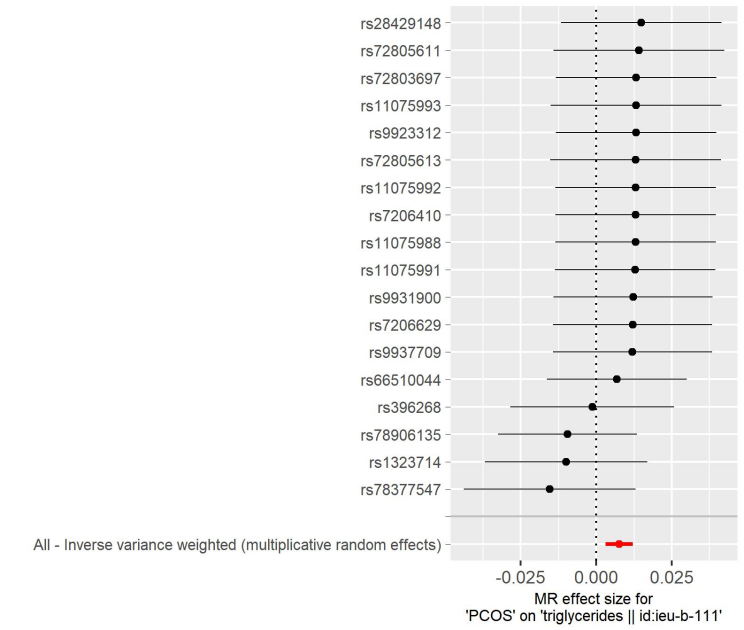


Figure S7. Funnel plot of causal associations between PCOS and lipid traits.

A: HDL and PCOS


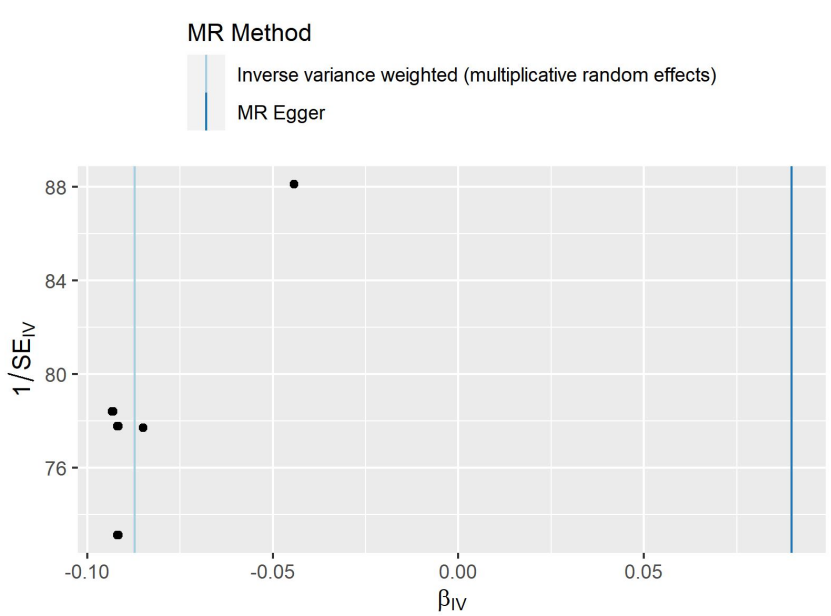


B: LDL and PCOS


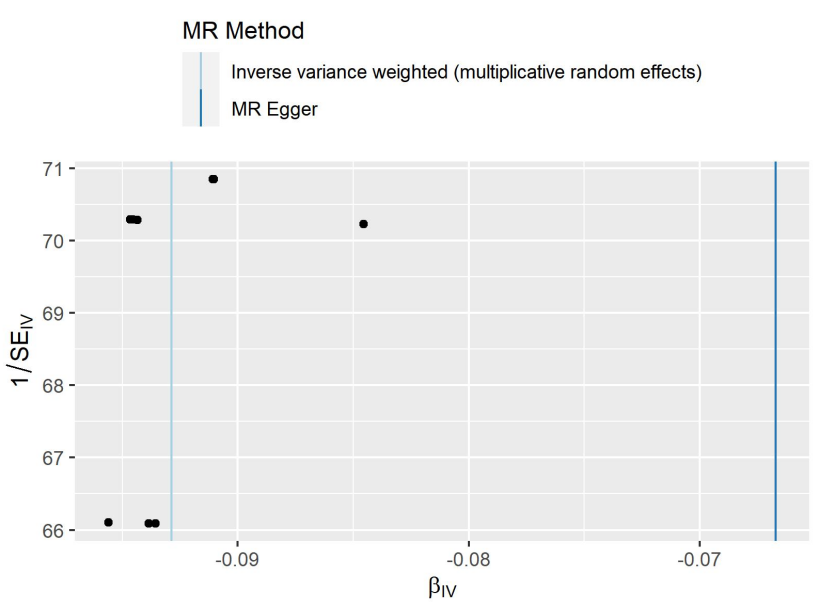


C: TG and PCOS


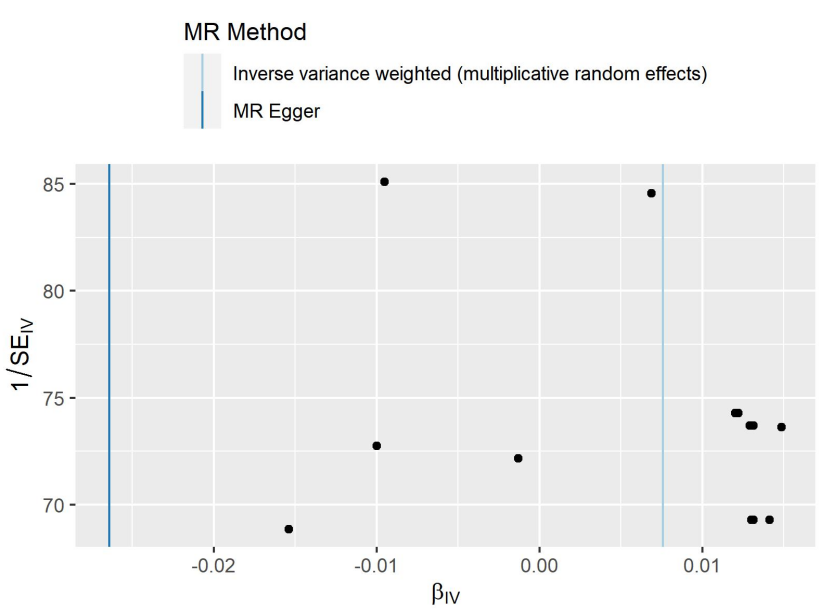


Figure S8. Leave-one-out plot to assess if a single variant is driving the associations between PCOS and lipid traits.

A: HDL and PCOS


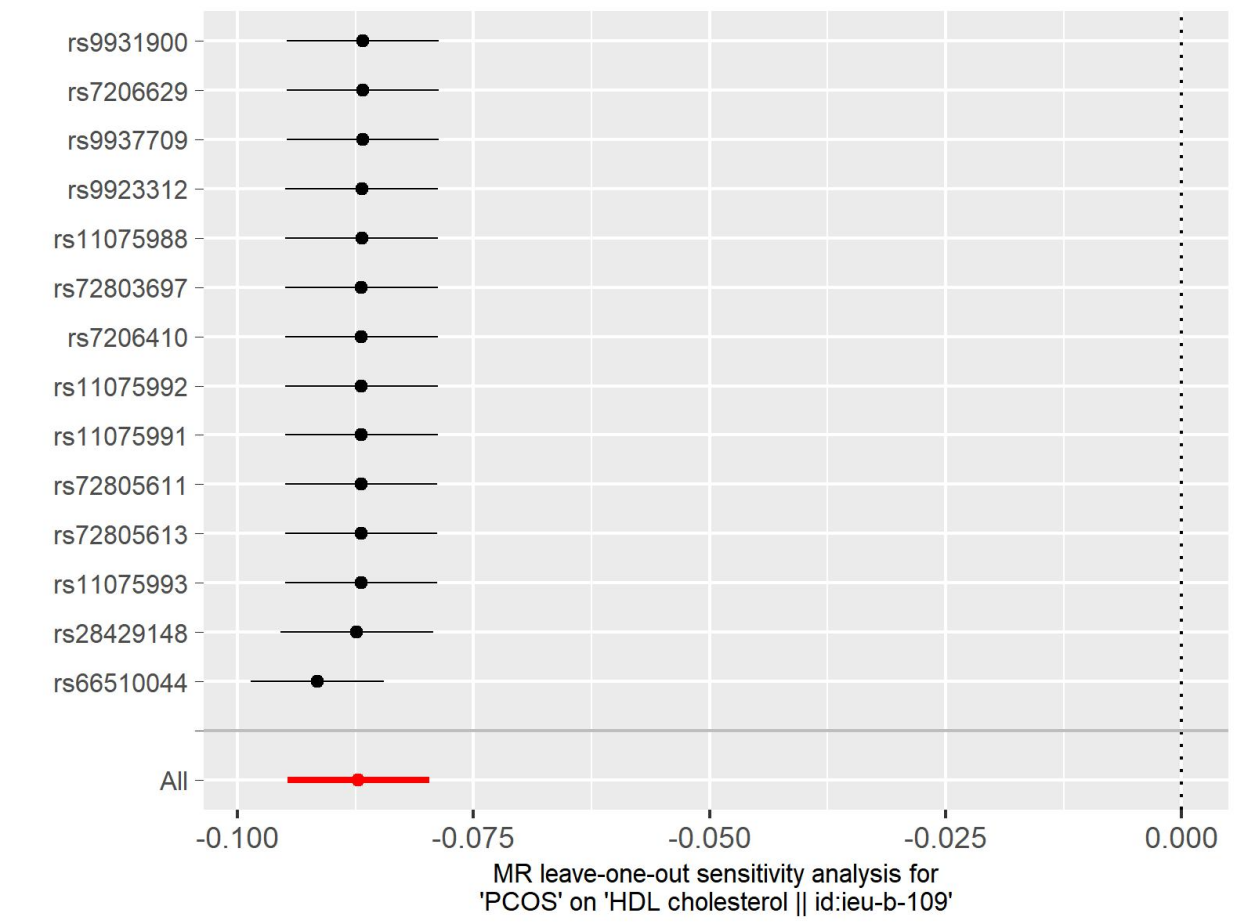


B: LDL and PCOS


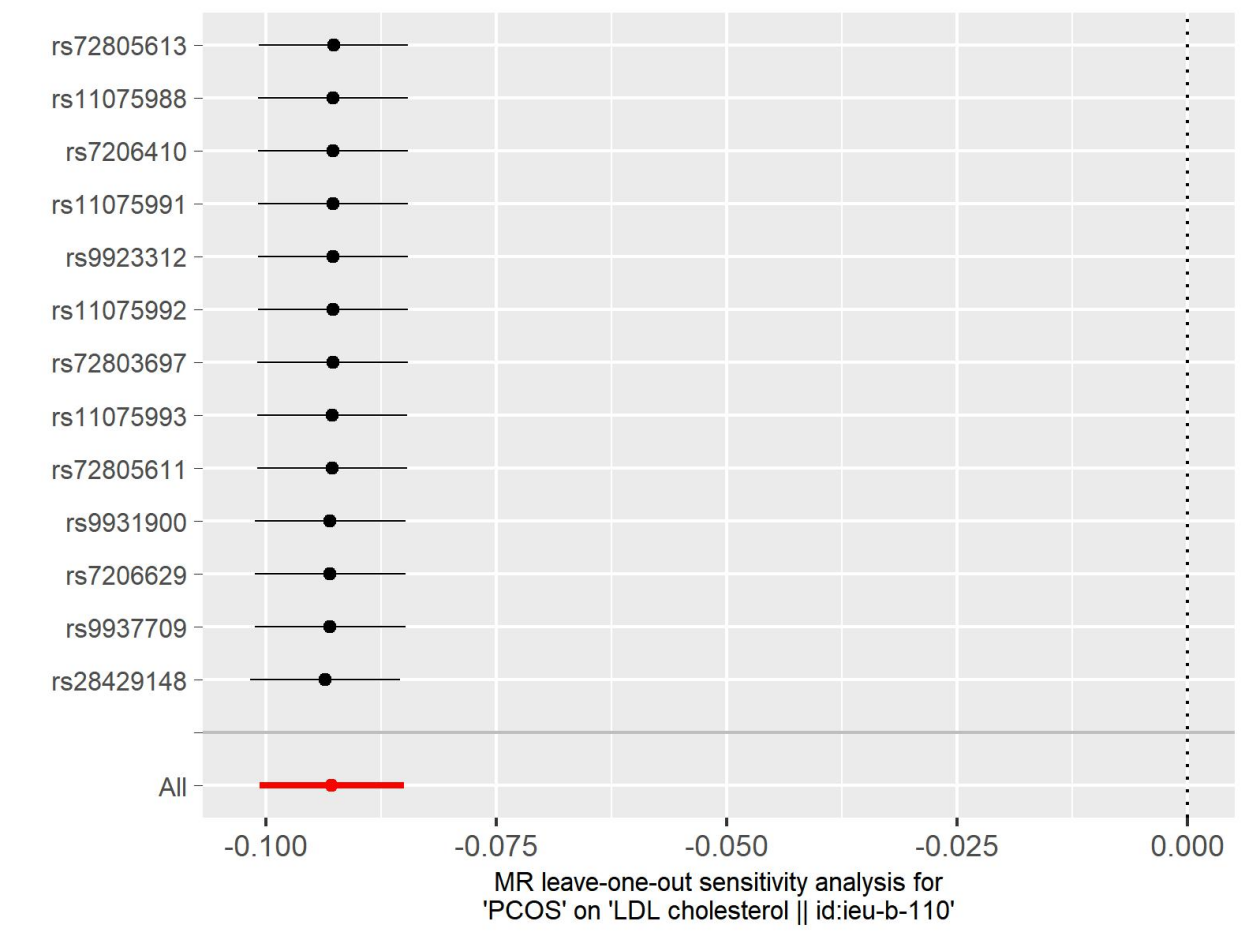


C: TG and PCOS


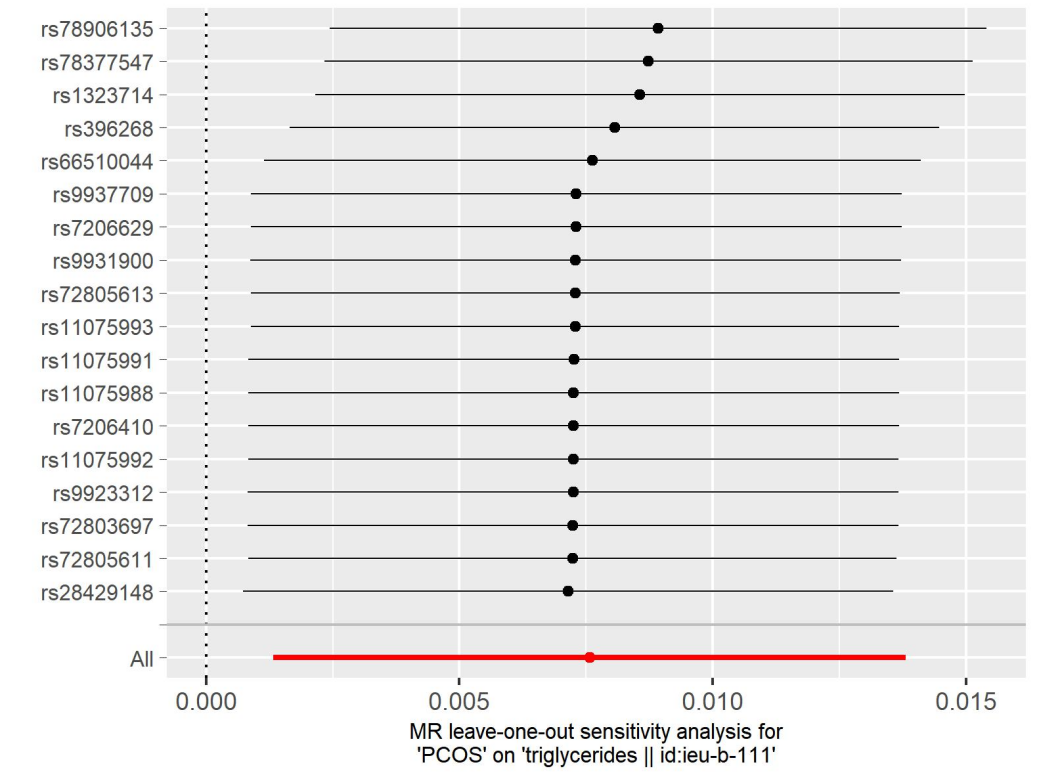


Supplementary Figure S9.

(A) Body weights of rats were recorded daily (n = 5 per group).

(B) Estrous cycles were monitored 14 days after treatment (n = 5). X-axis: Days; Y-axis: P (Proestrus, follicular development), E (Estrus, ovulation and receptivity), M (Metestrus, corpus luteum formation), D (Diestrus, progesterone dominance/regression).

(C) Representative ovarian histology images stained with hematoxylin and eosin (H&E) (scale bar = 500 μm).

(D, E, H) Serum levels of LH, FSH, testosterone, PCSK9, and lipid profiles (TG, TC, HDL-C, LDL-C) were measured using commercial ELISA kits.

(F, G) Intestinal SURF4 expression was assessed at both mRNA and protein levels using qPCR and immunohistochemistry, respectively.

(I) Pearson correlation analysis revealed a strong positive correlation between intestinal SURF4 expression and serum testosterone levels in the DHEA group (r = 0.884, p = 0.047), whereas no significant correlation was found in the Control group (r = 0.417, p = 0.485).

Data are presented as mean ± standard error of the mean (SEM). *p < 0.05, **p < 0.01, ****p < 0.0001.


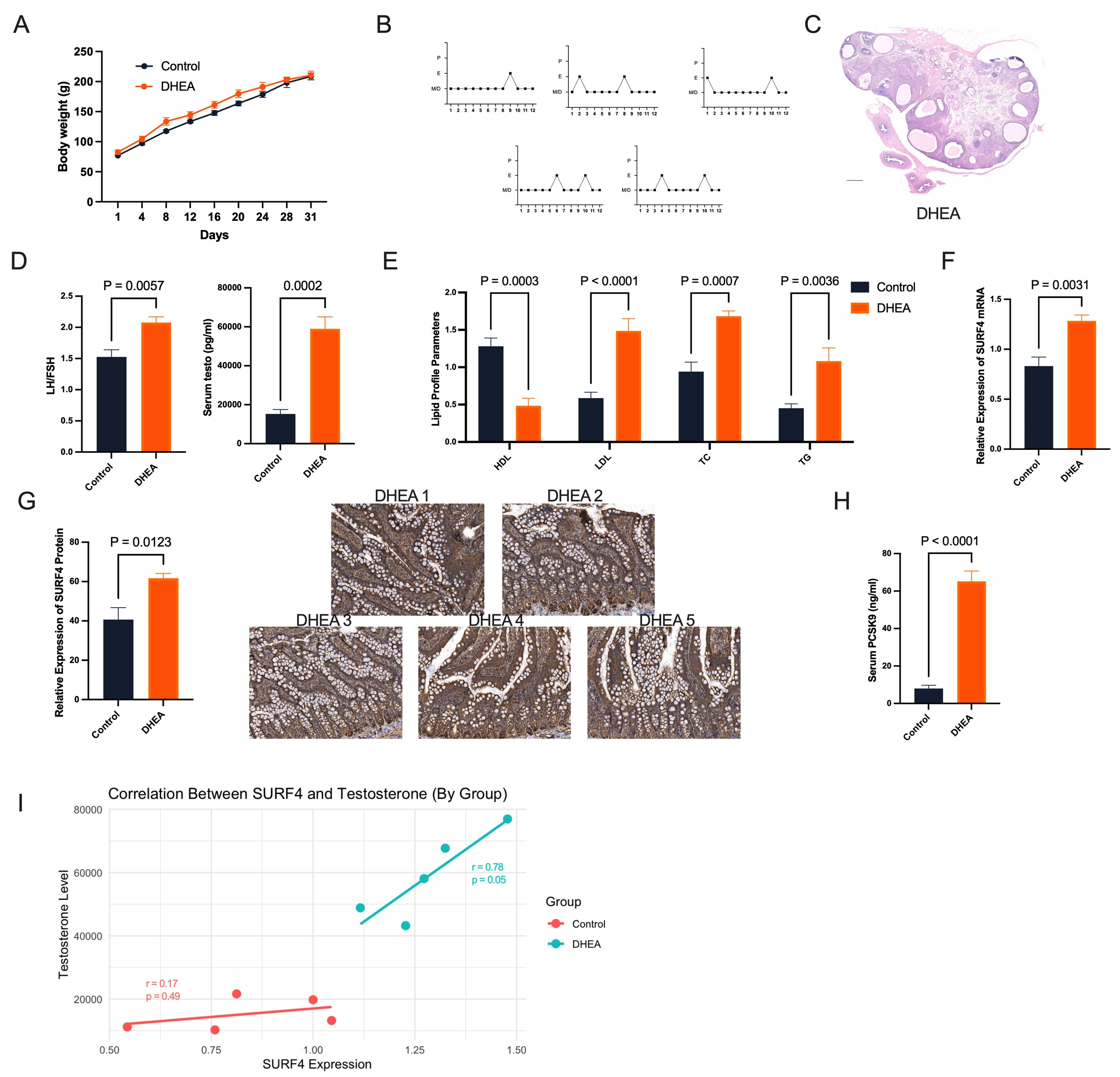

Supplement: Supplementary file 1 [file Supplementary_file_1.docx]
